# Supplementary material for: Cobolt: integrative analysis of multimodal single-cell sequencing data
Source: Genome Biol. 2021 Dec 28;22:351. doi: 10.1186/s13059-021-02556-z (PMC8715620; doi:10.1186/s13059-021-02556-z)
Supplement: Supplementary file 1 — Additional file 1 Supplementary Figures. PDF file containing Supplementary Figures referenced in the main text. [file 13059_2021_2556_MOESM1_ESM.pdf]

Supplementary Figures

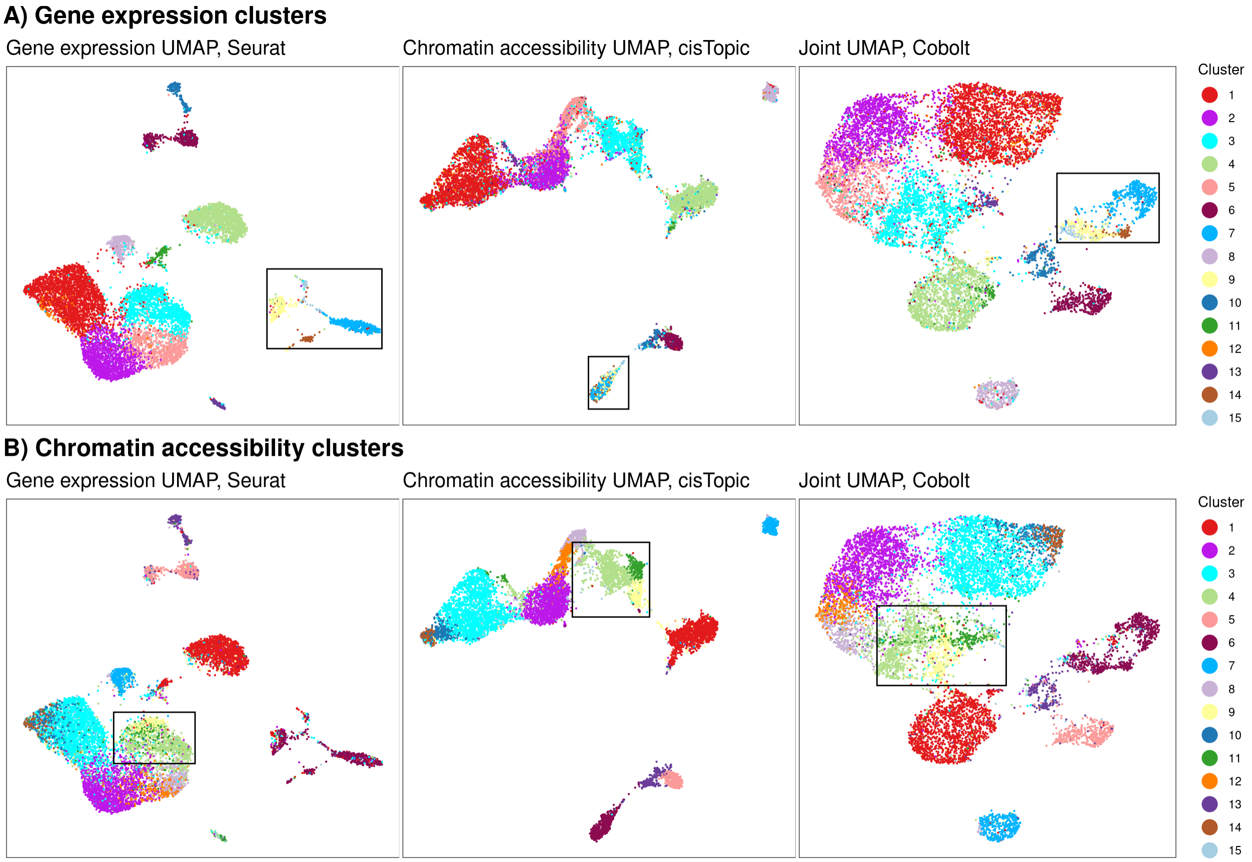

Fig. S1: Plotted are UMAP visualizations of the reduced dimensionality space created by analyzing only gene expression data using Seurat [Stuart et al., 2019b] and only chromatin accessibility using cisTopic [González-Blas et al., 2019], and jointly using both modalities (`Cobolt`). The cells are color-coded by the cluster they are assigned to based on clustering of A) gene expression modality and B) chromatin accessibility. We note that the cluster colors are randomly and separately assigned for panel A and B. In both cases, we see distinct clusters that are found in one modality but are not reflected in the other modality. Highlighted in the panels of A) are cells that show separated clusters in the gene expression, but not in chromatin accessibility; these cells show gene markers that show that these clusters correspond to the non-neuronal celltypes including astrocytes (Astro), microglial cells (MGC), oligodendrocyte precursors (OPC), and oligodendrocytes (Oligo) (See Fig S2A). Highlighted in B) are cells that are well separated in chromatin accessibility but not in gene expression, and are potentially a subtype of Layer 6 cells (see Fig S2B,C). Both sets of highlighted cells show separation in the joint analysis of `Cobolt`. In both these cases, the results of the `Cobolt` analysis shows that `Cobolt` integrates both modalities to find these modality-specific subtype differences: the two highlighted cell populations mentioned above are both distinguishable in the `Cobolt` reduced dimensions, and their cluster median silhouette widths are improved compared to single modality analysis (Fig S3). These results demonstrate the power of a joint analysis of modalities using `Cobolt` for a comprehensive understanding of subtypes.

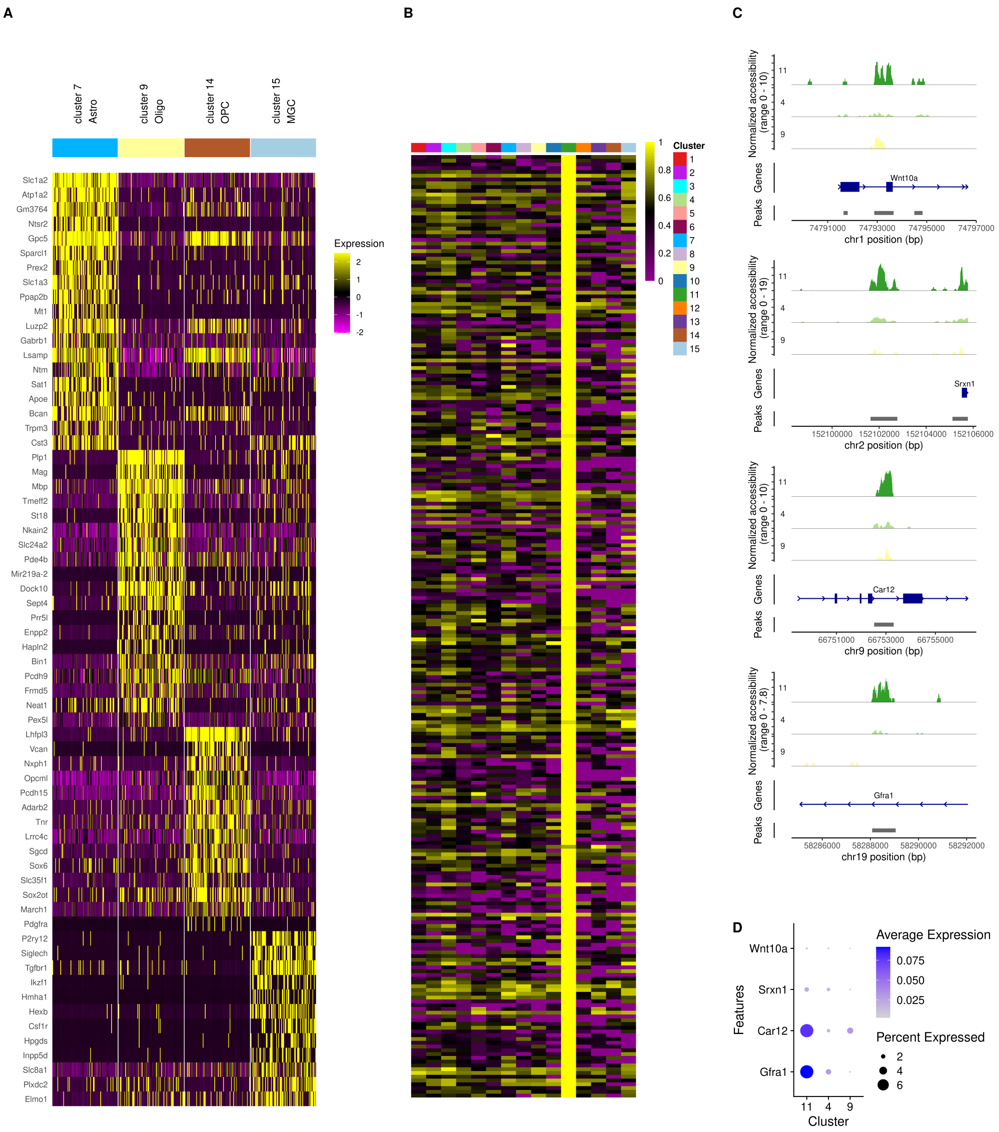

Fig. S2: **Clusters of SNARE-seq found by `Cobolt` and not found in one of the single modality analyses**. (A) Top genes from a differential expression analysis performed on clusters mentioned in the text as correctly identified by `Cobolt`. The cluster colors correspond to the mRNA clusters colored in Fig S1A found by clustering the mRNA expression only using Seurat (i.e. not using the joint representation of `Cobolt`). The clustering of only the mRNA expression from the SNARE-seq data distinguishes clusters 7, 9, 14, and 15 (blue, yellow, brown and light blue) which are not apparent in the chromatin accessibility data, but are distinguished by `Cobolt`. *Atp1a2, Bcan*, and *Cst3* are highly expressed in cluster 7 (blue) and are markers of astrocytes (Astro) [Zeng et al., 2012]; *Mbp* is highly expressed in cluster 9 (yellow) and a marker of oligodendrocytes (Oligo); *Pdgfra* is highly expressed in cluster 14 (brown) and is a marker of oligodendrocyte precursors (OPC) [Yao et al., 2021]; and *Siglech* and *P2ry12* are highly expressed in cluster 15 (light blue) and are markers of microglial cells (MGC) [Tasic et al., 2018]. (B, C, D) Clustering of only the chromatin accessibility from the SNARE-seq data (Fig S1B) discovers cluster 11 (green) which is not apparent in the mRNA expression clusters, but is found by `Cobolt`. Plotted in (B) are normalized accessibility levels of the top 200 DA peaks between cluster 11 (dark green) and its nearby cluster 4 and 9. Cluster 4, 9, and 11 are all identified as Layer 5/6 cells from their gene expression profile. We show examples of top peaks that are close to genes in (C) and the corresponding gene expression levels in (D), where *Car12* is a marker gene of Layer 6 subtype L6 Car12 Tasic et al. [2016].

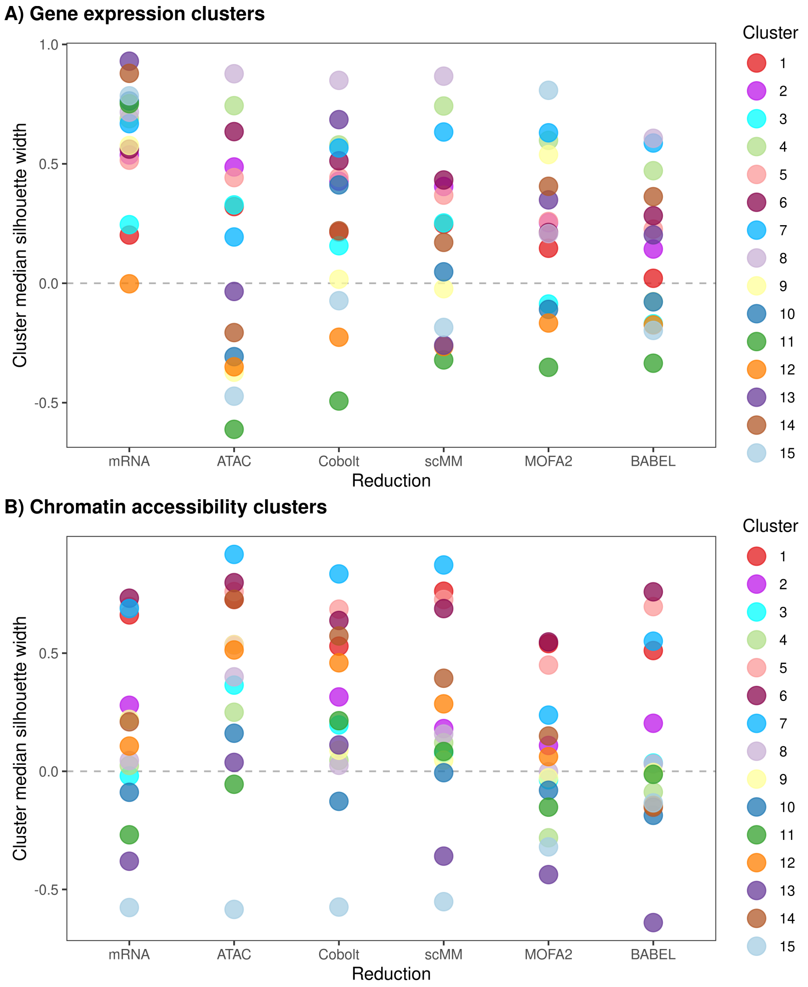

Fig. S3: The median silhouette width of SNARE-seq clusters generated using A) gene expression modality and B) chromatin accessibility. For each of the clustering, we calculate the median silhouette width per cluster on reduced dimensionality space created by analyzing only gene expression data (using Seurat), only chromatin accessibility (using cisTopic), and jointly with both modalities using `Cobolt`, scMM, MOFA2, and BABEL. Cluster colors are assigned the same as in Fig S1.

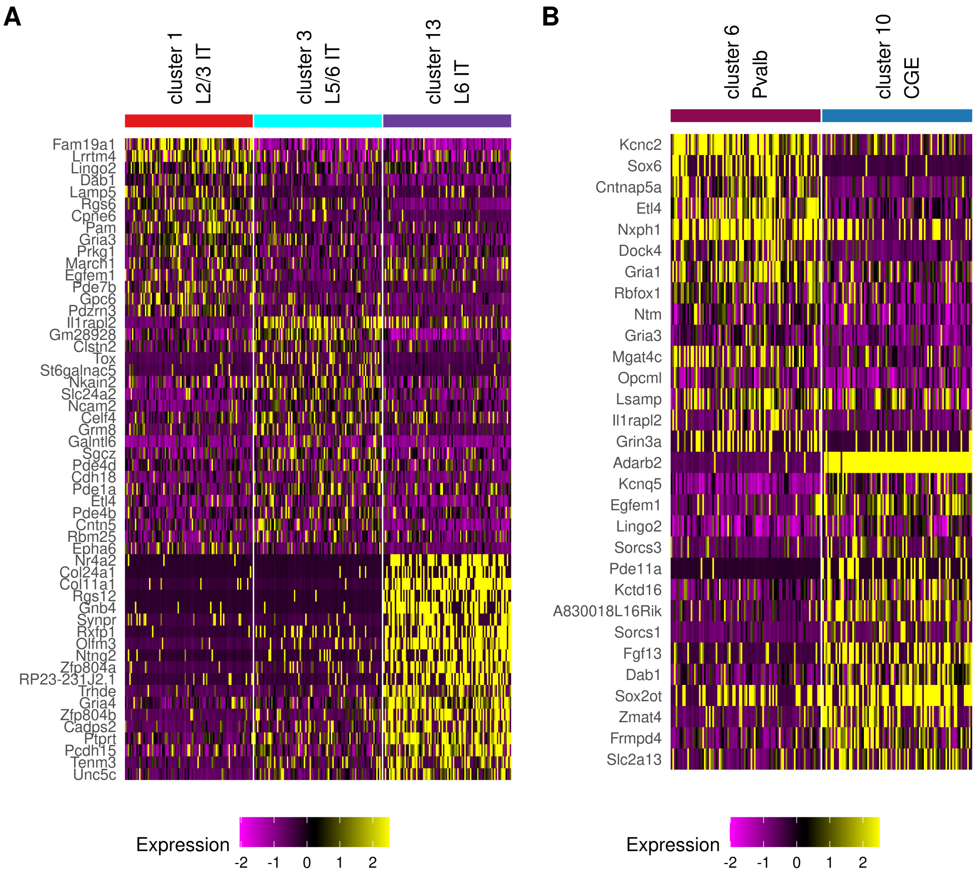

Fig. S4: **Clusters of SNARE-seq found by `Cobolt` and not found by other multi-modality methods**. Each heatmap shows top genes from a differential expression analysis performed on clusters mentioned in the text as correctly identified by `Cobolt` but missed by another multi-modality method. The cluster colors correspond to the mRNA clusters colored in Figure 2A and Fig S1A which were found by clustering only the mRNA expression using Seurat (i.e. not using the joint representation of `Cobolt`). (A) `Cobolt` distinguishes the cells in cluster 13 (purple) from cells in the surrounding clusters 1 (red) and 3 (cyan) which scMM does not distinguish. Cluster 13 shows strong expression of *Col24a1, Gnb4, Rxfp1, Nr4a2, Ntng2, Car3* which are known markers of Layer 6 cells (B) `Cobolt` distinguishes the cells in cluster 5 (mauve) from cells in cluster 9 (blue) which MOFA2 does not distinguish. Cluster 5 shows expression of Sox6, a marker of Pvalb cells while cluster 9 shows expression of Adarb2, a marker of CGE cells [Yao et al., 2021].

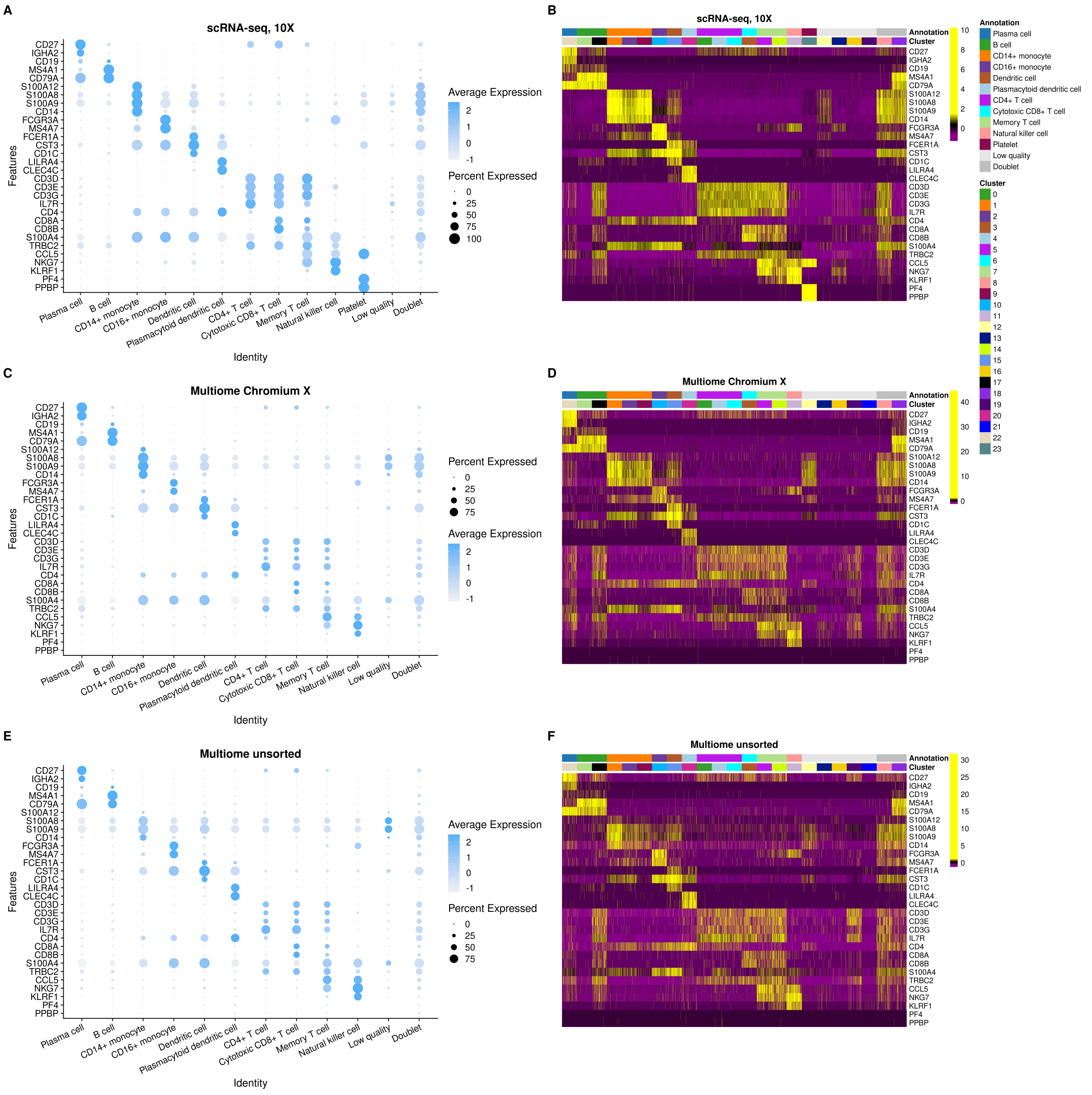

Fig. S5: **Expression of known marker genes of PMBCs, mRNA Expression** These plots show the mRNA expression of known marker genes of common cell types expected in PMBCs [Ding et al., 2020, Stuart et al., 2019a, Pliner et al., 2019, Franzén et al., 2019] for cells from the three PBMC mRNA expression datasets: (A-B) scRNA-seq, (C-D) Multiome Chromium X and (E-F) Multiome unsorted. The heatmaps (right) show the expression of cells for these gene markers in the clusters identified by the clustering of the `Cobolt` integration of the PBMC datasets. The results of clustering the `Cobolt` integration of the PBMC datasets are indicated above the cells; also indicated above are our assignment of these clusters to the known cell types based on these markers. The dot plots (left) show the expression of these gene markers for cells based on their assignment of to these known celltypes, illustrating that the assignments of clusters to known cell types result in cell types that follow known patterns in these gene markers.

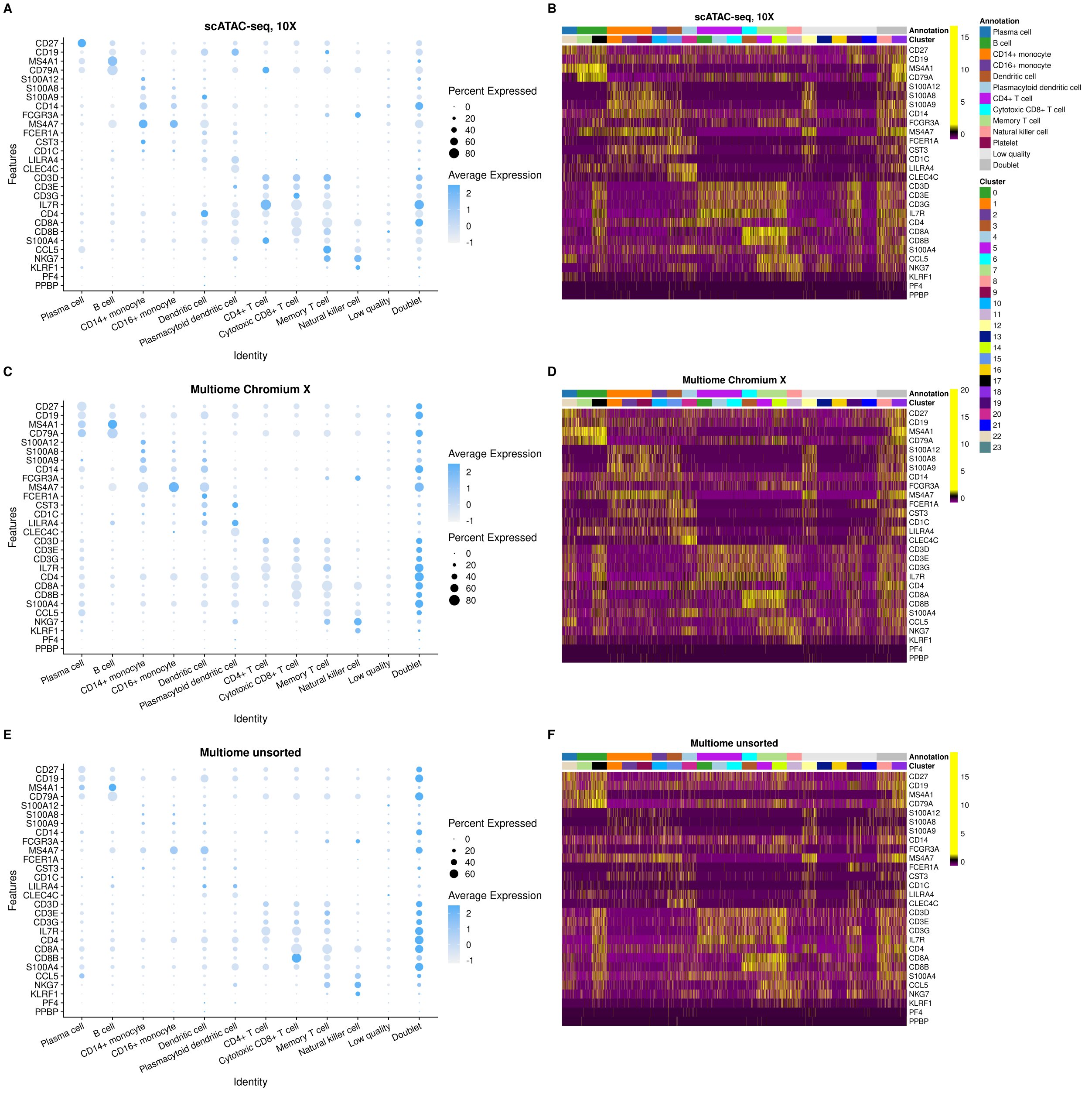

Fig. S6: **Expression of known marker genes of PMBCs, Chromatin Accessibility** These plots show the chromatin accessibility of known marker genes of common cell types expected in PMBCs [Ding et al., 2020, Stuart et al., 2019a, Pliner et al., 2019, Franzén et al., 2019] for cells from the three PBMC chromatim accessibility datasets: (A-B) scATAC-seq, (C-D) Multiome Chromium X and (E-F) Multiome unsorted. Chromatin accessibility levels are summarized over gene body and promoter regions. See Fig S5 for futher details.

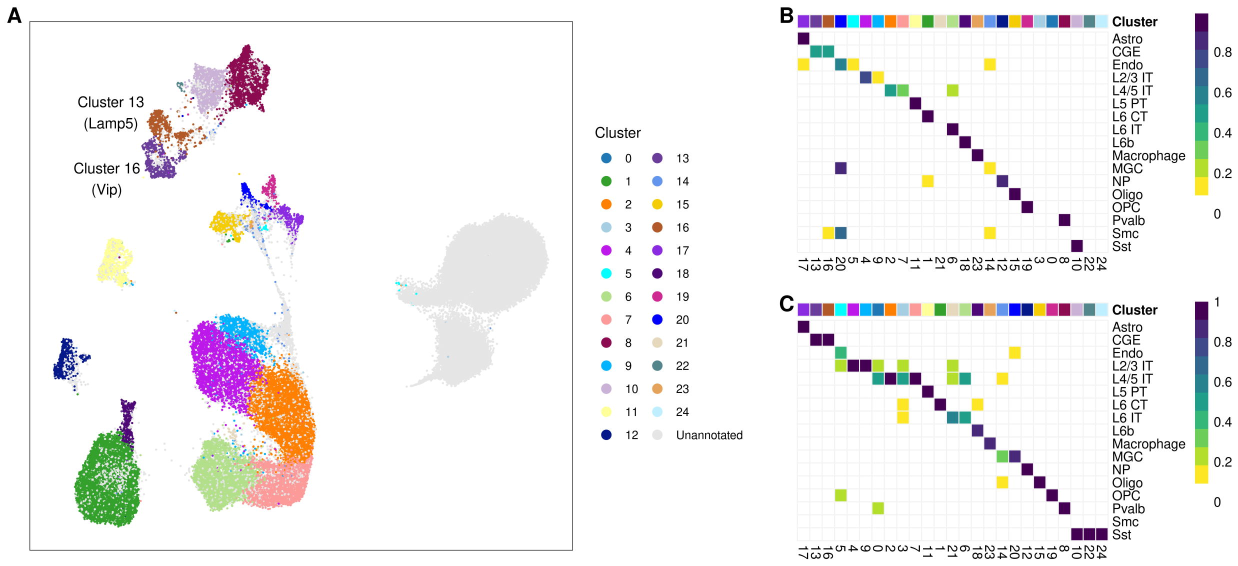

Fig. S7: `Cobolt` **clustering results on the mouse cortex data integration with cluster 13 and cluster 16 discussed in Figure 4 highlighted.** A) UMAP visualization colored by clusters. B, C) Concordance matrices between clustering results and annotations, scaled to sum 1 by (B) rows and (C) columns.

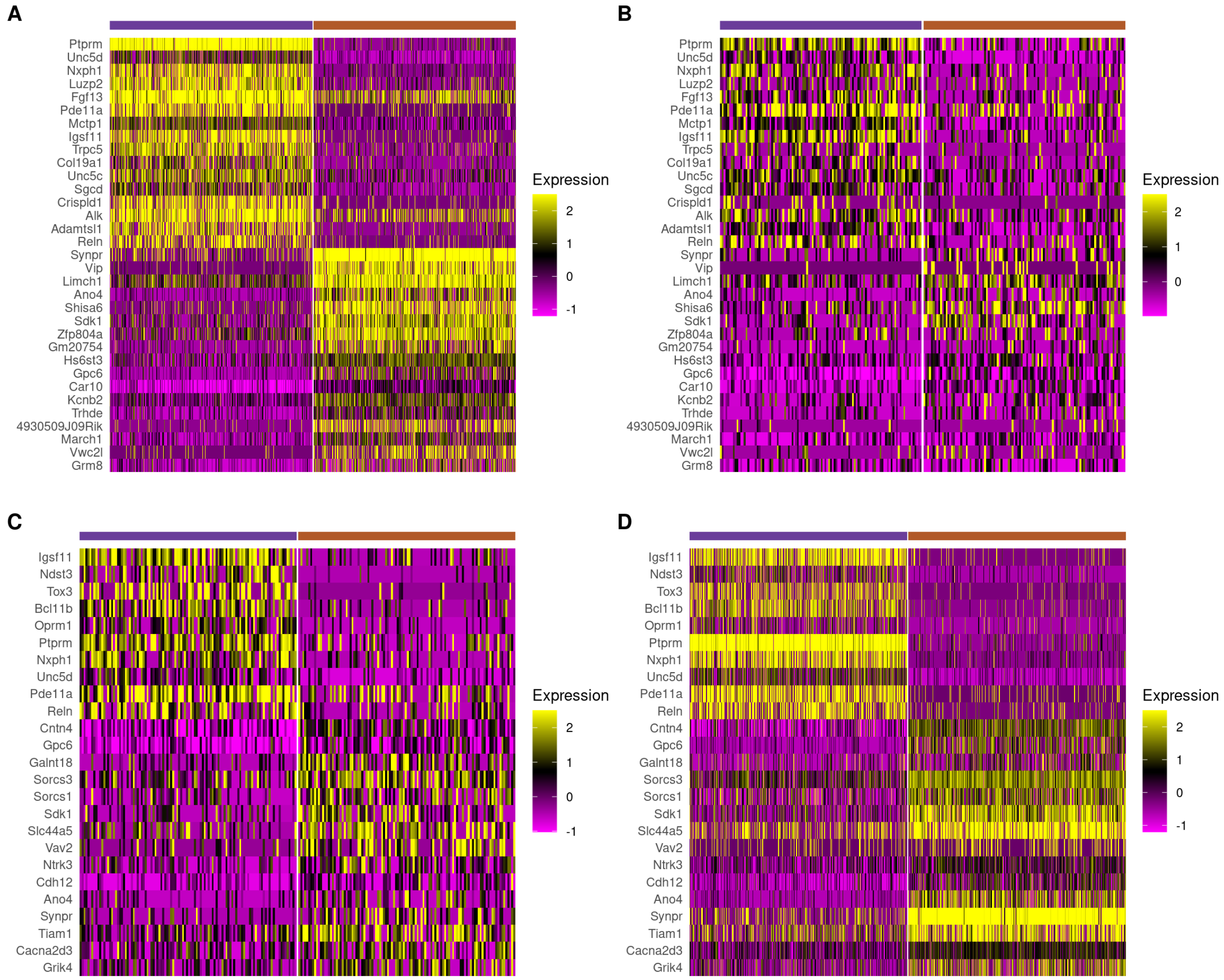

Fig. S8: **The DE and DA results between the *Vip* (cluster 16, brown) and the *Lamp5* (cluster 13, purple) clusters from the mouse cortex analysis.** A) The mRNA expression of the top DE genes between clusters 13 and 16. B) The ATAC chromatin accessibility levels of the genes identified in the DE analysis (shown in A)). C) The levels of ATAC chromatin accessibility of the top DA genes between clusters 13 and 16. D) The mRNA expression of the genes identified in the DA analysis (shown in C)). We note that all the top genes have the same direction of fold changes in the scRNA-seq and the scATAC-seq data, i.e., the genes with lower/higher gene expression in cluster 13 compared to cluster 16 are also less/more accessible.

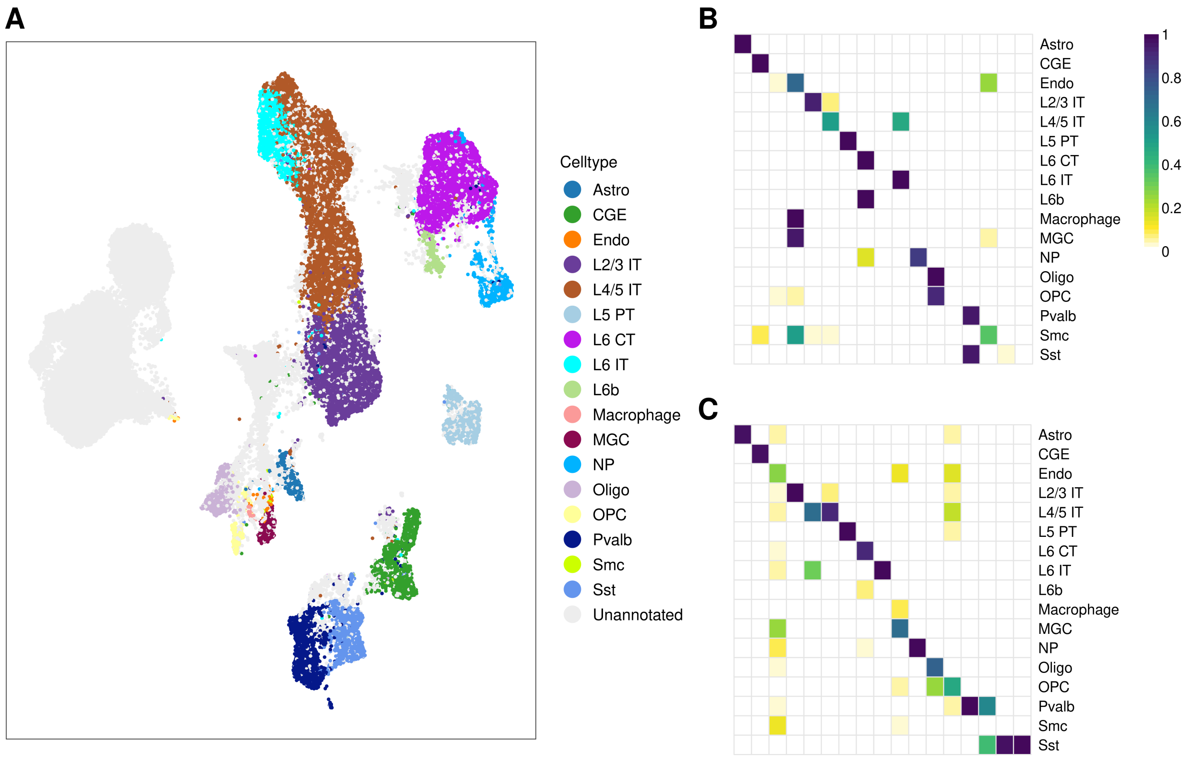

Fig. S9: `Cobolt` **results on the mouse cortex data integration with no filtering on cells and minimal filtering on features.** Genes and peaks with total counts larger than 10 and non-zero counts larger than 5 are retained in the model. A) UMAP visualization colored by cell type annotation. B, C) Concordance matrices between clustering results and annotations, scaled to sum 1 by (B) rows and (C) columns.

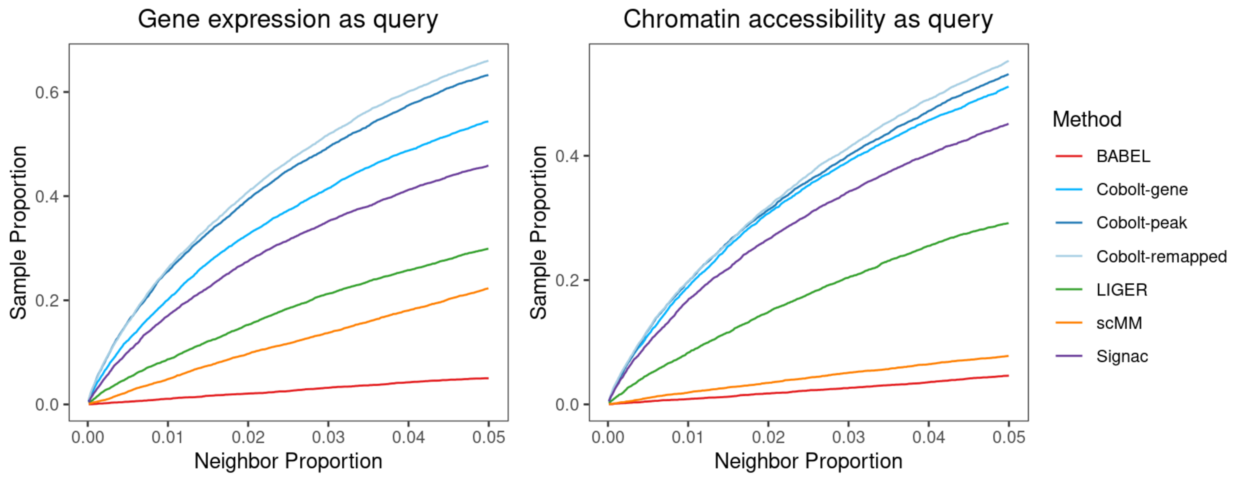

Fig. S10: **Full results of Figure 5A in the paper (cortical cells)**. Also included is a comparison of `Cobolt` dimensionality reduction on SNARE-seq data 1) using peaks called on the SNARE-seq (`Cobolt`-peak), 2) mapping counts to the peaks called on the MOp scATAC-seq (`Cobolt`-remapped), and 3) summarizing chromatin accessibility counts to gene regions (`Cobolt`-gene). No significant performance loss is observed for `Cobolt`-remapped compared to `Cobolt`-peak, indicating our strategy of mapping data to peaks called on similar datasets a simple but effective alternative in integration analysis. Using peaks vastly improved the performance relative to using the gene activities summarization (`Cobolt`-gene), suggesting that there is a loss of information in using gene summaries, such as distant binding sites not included in the gene summarization.

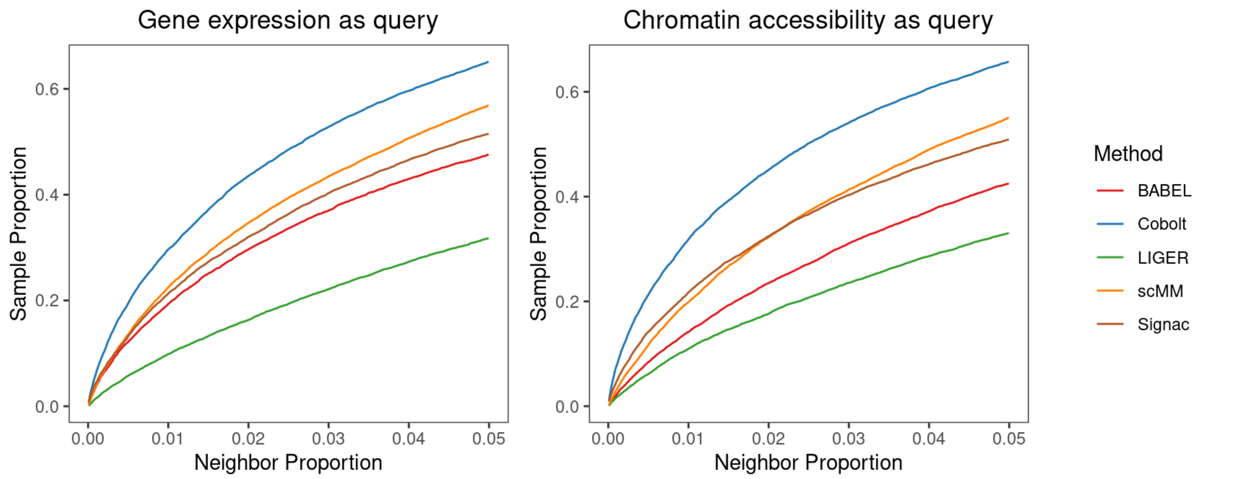

Fig. S11: Full results of Figure 5B in the paper (PBMC cells).

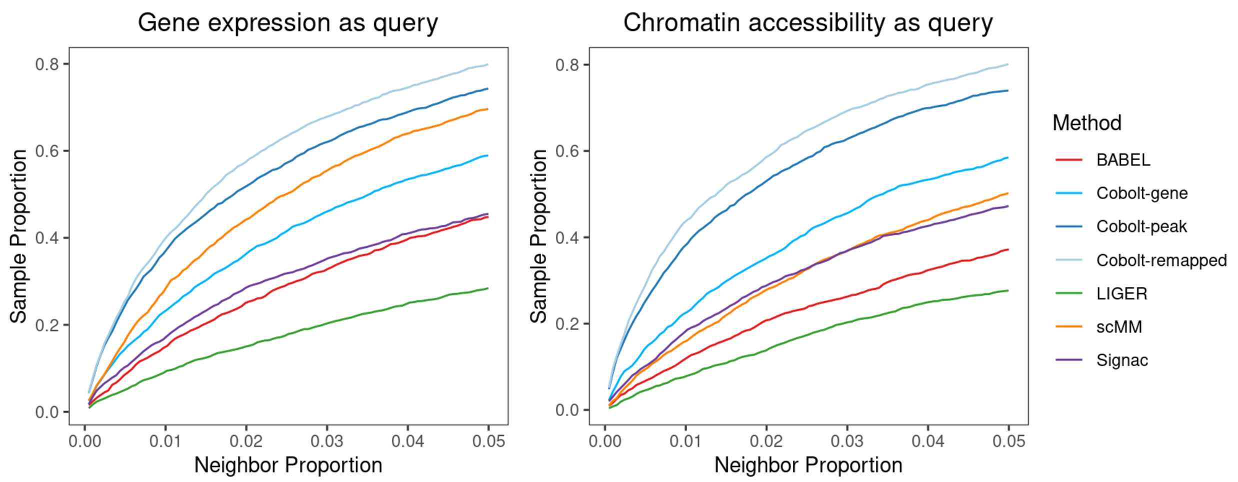

Fig. S12: An analysis as in Figure 5A in the paper, with a larger percentage of cells used as paired data in the training of scMM, BABEL, and Cobolt (80% rather than 20%). The x-axis shows the number of neighbors considered (k) as a proportion of the total testing sample size. The y-axis shows the proportion of cells whose paired data are within their k-nearest neighbors in the other modality.

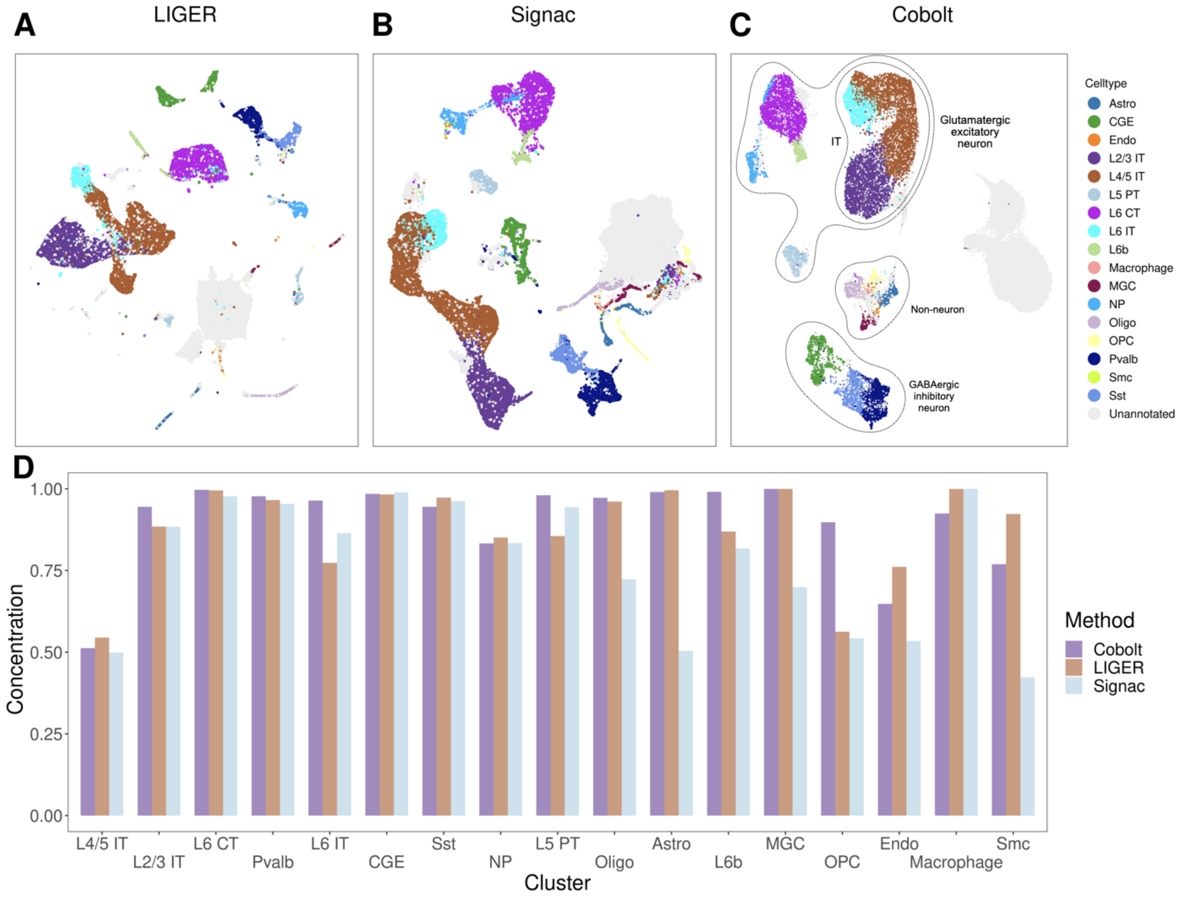

Fig. S13: A, B, C) UMAP visualizations generated by (A) LIGER, (B) Signac, and (C) `Cobolt` aligning MOp scATAC-seq and scRNA-seq, colored by cell type annotations. The three major classes of cell types—GABAergic inhibitory neurons, glutamatergic excitatory neurons, and non-neurons—are indicated on the `Cobolt` UMAP plot. D) Barplot of maximum overlap proportion between identified clusters and each cell type. For the complete concordance between clustering results and *a priori* cell type labels, see Fig S14).

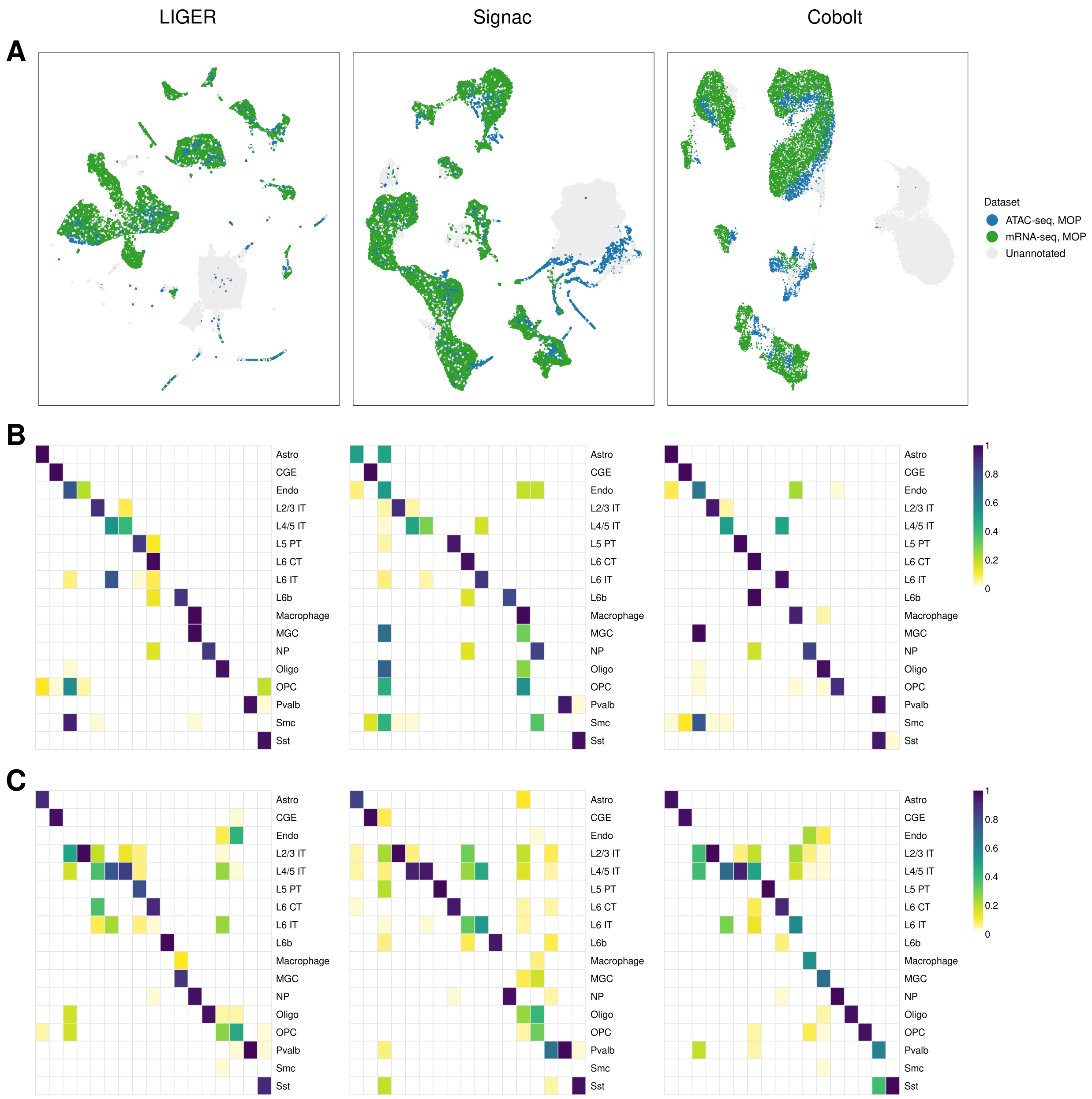

Fig. S14: A) UMAP visualizations generated by LIGER, Signac, and `Cobolt` for MOp scATAC-seq and scRNA-seq, colored by datasets. B, C) Concordance matrices between clustering results and annotations, scaled to sum 1 by (B) rows and (C) columns.

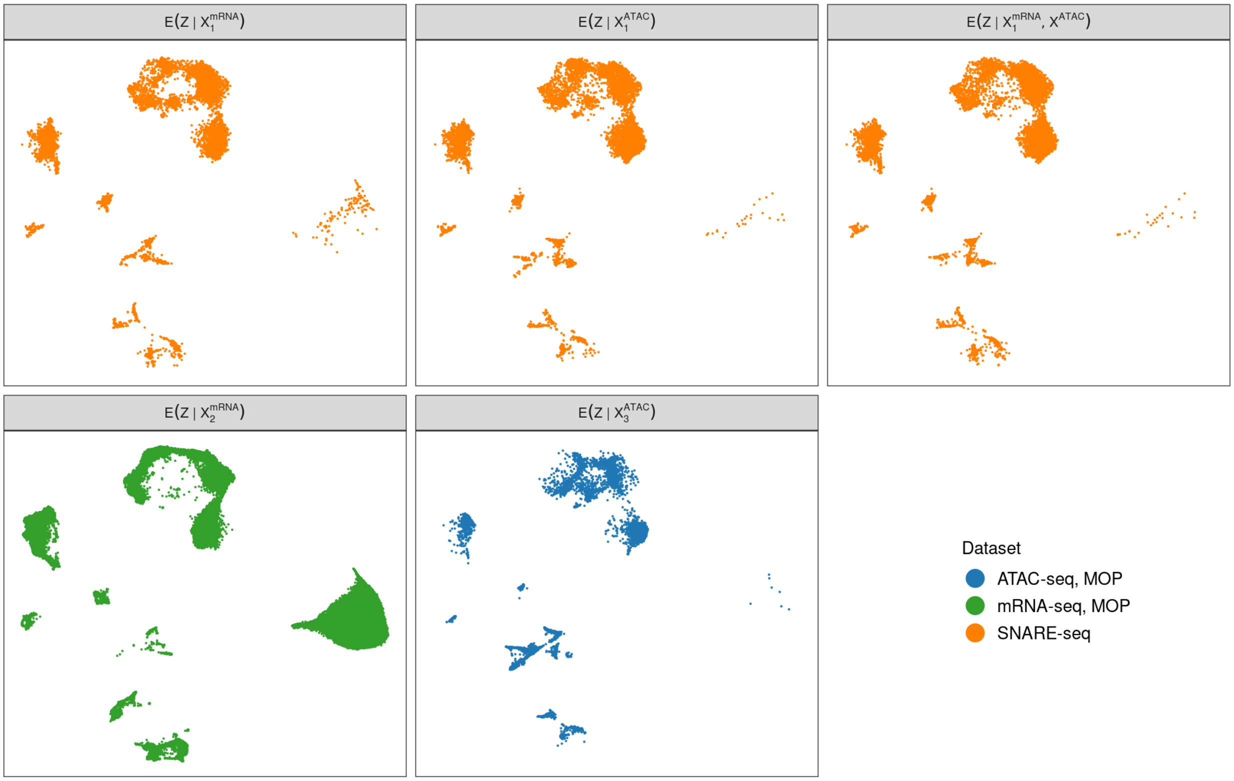

Fig. S15: **Estimates of the posterior means *without* missing modality correction.** The upper panel shows the UMAP dimensionality reduction plots of the posterior means $\hat{Z}_1 = E(Z|X_1^{mRNA}, X_1^{ATAC})$, $\hat{Z}_1^{mRNA} = E(Z|X_1^{mRNA})$, and $\hat{Z}_1^{ATAC} = E(Z|X_1^{ATAC})$ estimated for joint cells. The lower panel shows $\hat{Z}_2^{mRNA} = E(Z|X_2^{mRNA})$ and $\hat{Z}_3^{ATAC} = E(Z|X_3^{ATAC})$ estimated for single-modality cells. Other than the unannotated cells from MOp scRNA-seq data, cell populations from different datasets are well-aligned. We noticed slight distributional shifts between $\hat{Z}_1$, $\hat{Z}_1^{mRNA}$, and $\hat{Z}_1^{ATAC}$ in the upper panel, motivating additional correction procedures.

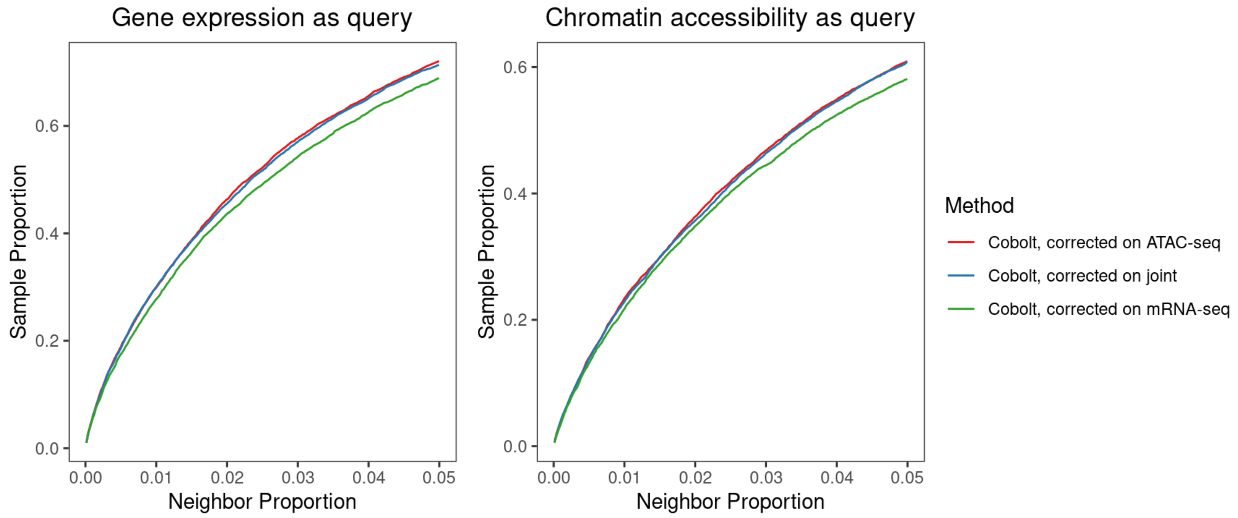

Fig. S16: **Effect of Prediction Space**. `Cobolt` was applied on the SNARE-seq data sets (similar to that of the Figure 5A) predicting coordinates into the ATAC-seq space $E(Z|X^{ATAC})$ (red), mRNA-seq space $E(Z|X^{mRNA})$ (green), or the joint space $E(Z|X_1^{mRNA}, X_1^{ATAC})$ (blue). Little difference is seen in the quality of the predictions in these different spaces.

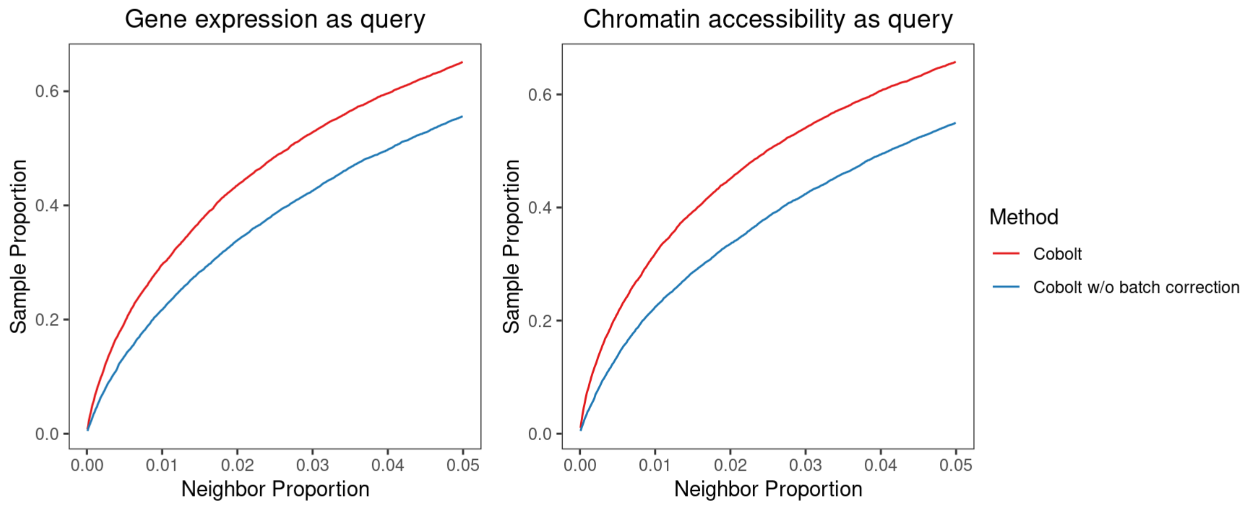

Fig. S17: **Effect of Batch Correction.** The effect of batch correction of the `Cobolt` model on the results. `Cobolt` was applied on the two 10X multiome data (similar to that of the Figure 5B) with and without the batch correction. The batch correction terms improve the performance as indicated by the closer distance between paired testing cells.

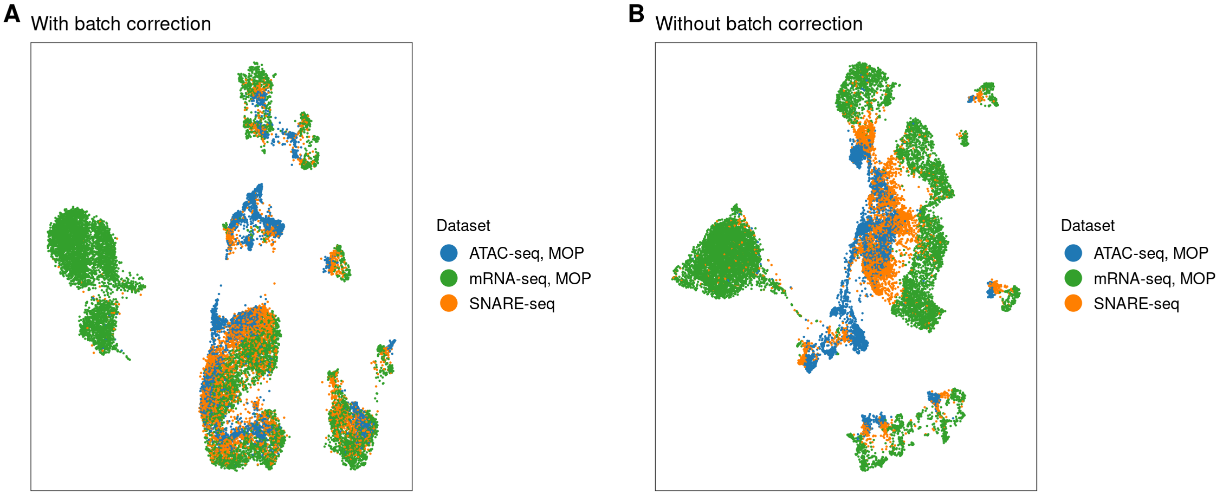

Fig. S18: **`Cobolt` on SNARE-seq data, with and without batch correction.** UMAP visualizations generated by applying `Cobolt` to the SNARE-seq data (A) with batch correction and (B) without batch correction.

References

Jiarui Ding, Xian Adiconis, Sean K Simmons, Monika S Kowalczyk, Cynthia C Hession, Nemanja D Marjanovic, Travis K Hughes, Marc H Wadsworth, Tyler Burks, Lan T Nguyen, et al. Systematic comparison of single-cell and single-nucleus rna-sequencing methods. *Nature biotechnology*, 38(6):737–746, 2020.

Oscar Franzén, Li-Ming Gan, and Johan LM Björkegren. Panglaodb: a web server for exploration of mouse and human single-cell rna sequencing data. *Database*, 2019, 2019.

Carmen Bravo González-Blas, Liesbeth Minnoye, Dafni Papasokrati, Sara Aibar, Gert Hulselmans, Valerie Christiaens, Kristofer Davie, Jasper Wouters, and Stein Aerts. cistopic: cis-regulatory topic modeling on single-cell atac-seq data. *Nature methods*, 16(5):397–400, 2019.

Hannah A Pliner, Jay Shendure, and Cole Trapnell. Supervised classification enables rapid annotation of cell atlases. *Nature methods*, 16(10):983–986, 2019.

Tim Stuart, Andrew Butler, Paul Hoffman, Christoph Hafemeister, Efthymia Papalexi, William M Mauck III, Yuhan Hao, Marlon Stoeckius, Peter Smibert, and Rahul Satija. Comprehensive integration of single-cell data. *Cell*, 177(7):1888–1902, 2019a.

Tim Stuart, Andrew Butler, Paul Hoffman, Christoph Hafemeister, Efthymia Papalexi, William M Mauck III, Yuhan Hao, Marlon Stoeckius, Peter Smibert, and Rahul Satija. Comprehensive integration of single-cell data. *Cell*, 177(7):1888–1902, 2019b.

Bosiljka Tasic, Vilas Menon, Thuc Nghi Nguyen, Tae Kyung Kim, Tim Jarsky, Zizhen Yao, Boaz Levi, Lucas T Gray, Staci A Sorensen, Tim Dolbeare, et al. Adult mouse cortical cell taxonomy revealed by single cell transcriptomics. *Nature neuroscience*, 19(2):335–346, 2016.

Bosiljka Tasic, Zizhen Yao, Lucas T. Graybuck, Kimberly A. Smith, Thuc Nghi Nguyen, Darren Bertagnolli, Jeff Goldy, Emma Garren, Michael N. Economo, Sarada Viswanathan, Osnat Penn, Trygve Bakken, Vilas Menon, Jeremy Miller, Olivia Fong, Karla E. Hirokawa, Kanan Lathia, Christine Rimorin, Michael Tieu, Rachael Larsen, Tamara Casper, Eliza Barkan, Matthew Kroll, Sheana Parry, Nadiya V. Shapovalova, Daniel Hirschstein, Julie Pendergraft, Heather A. Sullivan, Tae Kyung Kim, Aaron Szafer, Nick Dee, Peter Groblewski, Ian Wickersham, Ali Cetin, Julie A. Harris, Boaz P. Levi, Susan M. Sunkin, Linda Madisen, Tanya L. Daigle, Loren Looger, Amy Bernard, John Phillips, Ed Lein, Michael Hawrylycz, Karel Svoboda, Allan R. Jones, Christof Koch, and Hongkui Zeng. Shared and distinct transcriptomic cell types across neocortical areas. *Nature*, 563(7729):72–78, 2018. ISSN 14764687. doi: 10.1038/s41586-018-0654-5. URL `http://dx.doi.org/10.1038/s41586-018-0654-5`.

Zizhen Yao, Hanqing Liu, Fangming Xie, Stephan Fischer, Ricky S. Adkins, Andrew I. Aldridge, Seth A. Ament, Anna Bartlett, M. Margarita Behrens, Koen Van den Berge, Darren Bertagnolli, Hector Roux de Bézieux, Tommaso Biancalani, A. Sina Booeshaghi, Héctor Corrada Bravo, Tamara Casper, Carlo Colantuoni, Jonathan Crabtree, Heather Creasy, Kirsten Crichton, Megan Crow, Nick Dee, Elizabeth L. Dougherty, Wayne I. Doyle, Sandrine Dudoit, Rongxin Fang, Victor Felix, Olivia Fong, Michelle Giglio, Jeff Goldy, Mike Hawrylycz, Brian R. Herb, Ronna Hertzano, Xiaomeng Hou, Qiwen Hu, Jayaram Kancherla, Matthew Kroll, Kanan Lathia, Yang Eric Li, Jacinta D. Lucero, Chongyuan Luo, Anup Mahurkar, Delissa McMillen, Naeem M. Nadaf, Joseph R. Nery, Thuc Nghi Nguyen, Sheng-Yong Niu, Vasilis Ntranos, Joshua Orvis, Julia K. Osteen, Thanh Pham, Antonio Pinto-Duarte, Olivier Poirion, Sebastian Preissl, Elizabeth Purdom, Christine Rimorin, Davide Risso, Angeline C. Rivkin, Kimberly Smith, Kelly Street, Josef Sulc, Valentine Svensson, Michael Tieu, Amy Torkelson, Herman Tung, Eeshit Dhaval Vaishnav, Charles R. Vanderburg, Cindy van Velthoven, Xinxin Wang, Owen R. White, Z. Josh Huang, Peter V. Kharchenko, Lior Pachter, John Ngai, Aviv Regev, Bosiljka Tasic, Joshua D. Welch, Jesse Gillis, Evan Z. Macosko, Bing Ren, Joseph R. Ecker, Hongkui Zeng, and Eran A. Mukamel. A transcriptomic and epigenomic cell atlas of the mouse primary motor cortex. *Nature*, 598(7879):103–110, 2021. ISSN 0028-0836. doi: 10.1038/s41586-021-03500-8. URL `http://dx.doi.org/10.1038/s41586-021-03500-8`.

Hongkui Zeng, Elaine H. Shen, John G. Hohmann, Seung Wook Oh, Amy Bernard, Joshua J. Royall, Katie J. Glattfelder, Susan M. Sunkin, John A. Morris, Angela L. Guillozet-Bongaarts, Kimberly A. Smith, Amanda J. Ebbert, Beryl Swanson, Leonard Kuan, Damon T. Page, Caroline C. Overly, Ed S. Lein, Michael J. Hawrylycz, Patrick R. Hof, Thomas M. Hyde, Joel E. Kleinman, and Allan R. Jones. Large-scale cellular-resolution gene profiling in human neocortex reveals species-specific molecular signatures. *Cell*, 149(2):483–496, 2021/10/19 2012. doi: 10.1016/j.cell.2012.02.052. URL https://doi.org/10.1016/j.cell.2012.02.052.
