# Supplementary material for: Cobolt: integrative analysis of multimodal single-cell sequencing data
Source: Genome Biol. 2021 Dec 28;22:351. doi: 10.1186/s13059-021-02556-z (PMC8715620; doi:10.1186/s13059-021-02556-z)
Supplement: Supplementary file 5 — Additional file 5 Review history. [file 13059_2021_2556_MOESM5_ESM.docx]

Review History

**First round of review**

**Reviewer 1**

**Were you able to assess all statistics in the manuscript, including the appropriateness of statistical tests used?**

Yes: I have read the section and the supplementary materials for the statistical method. See my comments in the report

**Were you able to directly test the methods?**

No

**Comments to author:**

This paper introduces a new method Cobolt, which uses variational autoencoders to jointly align cells from single-modality sequencing data and multi-modality sequencing data. I think the method is in general thoughtful and well designed. Compared with other methods that can align cells from different modalities, Cobolt can make use of multi-modality sequencing data to help better align unpaired cells from different modalities. My main concern is that I feel the case studies are not convincing enough to show the usefulness and supreme of Cobolt, and the paper can be improved by trying Cobolt on more datasets.

Here are my comments:

1. Figure 2 and Figure S2 use the SNARE-seq data to show that Cobolt visualization can keep the cells separated for clusters that are unique in either the scRNA-seq data alone and scATAC-seq data alone. I think this is a nice comparison but my concern is that it's unclear whether these clusters are biologically meaningful cell subtypes that we hope to identify or not. For instance, is the small purple cluster in Figure 1A a real signal that we want to keep? In figure S2, do the dark green and light green clusters represent two real cell sub types? I have this concern as clustering and visualization results may change substantially if weuse different tuning parameters like resolution, number of PCs, number of nearest neighbors et al.

2. In Figure 3/4: seems the signals that are described in the main text are not easy to see from the figures, and the figures may need more clarifications. For instance, I did not find the Smc cells on the figure that is mentioned on page 6 line 39. Also, I'm not sure what clusters 13 and 16 are and where are they located so it's kind of hard to understand Fig 4A. I think the authors use Figure 4C to support the conclusion that subtypes of scATAC-seq cells are corrected identified after joint alignment. Since the scATAC-seq signals are weak, it would be more convincing if the authors can show more marker genes of the Lamp5 and Vip subtypes.

3. Cobolt aims to align cells from very distinct technologies and one concern for doing this is the batch effect across different platforms and datasets. Cobolt adjusts for batch effect by introducing a dataset specific B^{(i)}, however I feel that whether the batch correction works or not is not well evaluated in the case studies. The paper shows that the multi-modal data is helpful to better align the single-modal data by random splitting of the SNARE-seq cells. However, there is no batch differences here, so it would be nice if the authors can evaluate how robust Cobolt is when we anticipate batch effects across platforms.

4. For the variational autoencoder, based on my understanding, the authors assume that the posterior distribution of Z on cells with mutl-modal sequencing data (say (X^{(1)}, X^{(2)})) is a product of the posterior distribution of Z on each of the observed modality (X^{(i)}) (page 17). Will this hold only when marginally X^{(1)} and X^{(2)} are independent? So strictly speaking, is it true that the assumption on p(z|X^A) (page 17 lines 21-23) is actually never satisfied? Anyway, I think the autoencoder structure of Coboit is a nice design.

5. I'm still a bit confused about the assumption on p(X|Z). Seems that X are counts and authors assume that each entry of X follow a multinomial distribution. I do not quite understand why we can assume a multinomial distribution of X.

6. What are the chosen values for the hyperparameters in equations S1 and S2?

6. There seems to be a typo on page 10 line 25, the last word should be "encoders" instead of "decoders"?

**Reviewer 2**

**Were you able to assess all statistics in the manuscript, including the appropriateness of statistical tests used?**

No

**Were you able to directly test the methods?**

Yes

**Comments to author:**

The authors proposed a method named Cobolt trying to integrate multiple single-cell multi-modal data that were measured with different level of modalities. The method details were well presented. The method has been validated on two data sets (of one experimental setting) and compared with several existing methods. I have several concerns about this manuscript:

1) The author did not develop the original MVAE model, instead, they directly applied it on the task of integrate multiple single-cell multi-modal data without examining the property of MVAE and other VAE models designed for data of two or multiple modalities. This much lower the computational originality of this work. The advantage of MVAE is to handle datasets of a high number of modality, which have different level of missing measures on certain modalities. However, in single cell multi-omics data, normally joint analysis are only needed for two modalities (transcriptome and chromatin accessibility). There are several VAE models enabled the bidirectional generative models other than MVAE, such as JMVAE. The authors are expected to evaluate the most property model for single cell multi-omics data. Specifically, the MVAE assumes conditional in dependency of the modalities. However, this seems contradict to the underlying systems biology of transcriptional regulation. Gene expression and chromatin accessibility cannot be independent conditional to the low dimension latent variable z, which characterize the phenotypic characteristics of the cell, because chromatin accessibility is a very strong transcriptional regulatory signal that can directly affect the gene expression. Hence the conditional independence assumption of MVAE model could be fundamentally problematic for integrating scRNA-seq and scATAC-seq data.

2) A few similar works were published recently:

Chunman Zuo, Luonan Chen, Deep-joint-learning analysis model of single cell transcriptome and open chromatin accessibility data, Briefings in Bioinformatics, Volume 22, Issue 4, July 2021, bbaa287

Wang, T., Shao, W., Huang, Z. et al. MOGONET integrates multi-omics data using graph convolutional networks allowing patient classification and biomarker identification. Nat Commun 12, 3445 (2021).

3) VAE based models can hardly provide a deeper mining of biological functions. As presented in the manuscript, the current utilization of this method is only to improve cell clustering analysis. Cell clustering is a relatively well solved task in single cell data analysis. Mining of more biological insights are expected.

**Authors Response**

**Point-by-point responses to the reviewers’ comments:**

Reviewer 1

Critique: "This paper introduces a new method Cobolt, which uses variational autoencoders to jointly align cells from single-modality sequencing data and multi-modality sequencing data. I think the method is in general thoughtful and well designed. Compared with other methods that can align cells from different modalities, Cobolt can make use of multi-modality sequencing data to help better align unpaired cells from different modalities. My main concern is that I feel the case studies are not convincing enough to show the usefulness and supreme of Cobolt, and the paper can be improved by trying Cobolt on more datasets."

*Response: To further show the usefulness of Cobolt, we have added an analysis of an additional collection of datasets: two 10X multiome datasets along with 10X mRNA-Seq and 10X ATAC-Seq data from human peripheral blood mononuclear cells (PBMCs). Unlike the MOp data, this data did not have accompanying annotation, so we clustered the cells and related these clusters back to known marker genes of the major cell types of the PBMCs. This demonstrates a typical ``full'' analysis, following a standard pipeline of determining celltypes, only now across the cells from all modalities.*

*Further, this additional dataset allowed for the quantitative comparison between different methods in Figure 5 to improve our analysis. Rather than randomly dividing the 10X multiome data into two parts, like we did with the SNARE-Seq data, we took advantage of the fact that we have two 10X multiome datasets available from two different donors, with different gender and age. We took the data from the earlier run (``Multiome unsorted") and treated the paired modalities as single modalities for our testing. In this way, we are able to more closely match reality, where the single modality data comes from separate experiments from the multi-modality data, yet still have ground truth for our comparison.*

Critique: "1. Figure 2 and Figure S2 use the SNARE-seq data to show that Cobolt visualization can keep the cells separated for clusters that are unique in either the scRNA-seq data alone and scATAC-seq data alone. I think this is a nice comparison but my concern is that it’s unclear whether these clusters are biologically meaningful cell subtypes that we hope to identify or not. For instance, is the small purple cluster in Figure 1A a real signal that we want to keep? In figure S2, do the dark green and light green clusters represent two real cell sub types? I have this concern as clustering and visualization results may change substantially if weuse different tuning parameters like resolution, number of PCs, number of nearest neighbors et al."

*Response: We have added additional biological analysis of these clusters. We have performed differential expression/accessibility analysis as well as comparing their expression of known gene markers. For example, we show that the small purple cluster in Figure 2A display strong markers of a layer 6 IT Car3 subset of cells, unlike the Layer 5/6 and Layer 2/3 cells surrounding it (Supplementary Figure 4). Similarly, the clusters found only in the mRNA in Supplementary figure S2A (now Supplementary Figure 1) show biomarkers for well-known non-neuronal cell types (astrocytes, microglial, and oligodendrocytes cells) as shown in the new Supplementary Figure 2, as well as mapping to cells with this annotation in the MOP scRNA-Seq data during the integrative analysis of \texttt{Cobolt}.*

*Similarly, we perform differential accessibility analysis and show that the green cluster found in chromatin in the Supplementary Figure S2B (now Supplementary Figure 1B) are Layer 5/6 cells with distinct chromatin accessibility peaks, including near the gene \textit{Car12}, which is a marker gene of a previously annotated subtype of Layer 6 (Supplementary Figure 2BC).*

Critique: "2. In Figure 3/4: seems the signals that are described in the main text are not easy to see from the figures, and the figures may need more clarifications. For instance, I did not find the Smc cells on the figure that is mentioned on page 6 line 39. Also, I’m not sure what clusters 13 and 16 are and where are they located so it’s kind of hard to understand Fig 4A."

*Response: Thank you for pointing this out. We do discuss small clusters that are hard to detect in Figure 3B. We have added text to the figure identifying major cell types, in addition to the color-code legend that was there previously.*

*For Figure 4 and the reference to clusters 13 and 16, we realize that the text was not sufficiently clear as to the results we were presenting. These are clusters based on de novo clustering of our joint representation, while Figure 3B shows cell types based on the published annotation from the MOP paper and related marker genes. We clarified this analysis in the text, and have added a Supplementary Figure 7 that shows the results of our de novo clustering with clusters 13 and 16 highlighted.*

Critique: "I think the authors use Figure 4C to support the conclusion that subtypes of scATAC-seq cells are corrected identified after joint alignment. Since the scATAC-seq signals are weak, it would be more convincing if the authors can show more marker genes of the Lamp5 and Vip subtypes."

*Response: We have updated Figure 4 to include more marker genes from the scRNA-Seq clusters as given in Tasic et al (2018). This demonstrates that the clusters we identify show biological meaning in both the scRNA-Seq and scATAC-Seq, in the sense that these markers differentiate between the cluster 13 and 16 that we found and are coherent between the mRNA and chromatin expression. We further performed a de novo analysis of differential expression (DE) and differential accessibility (DA) to find genes that differentiate these clusters, with a heatmap of the results shown in Supplemental Figure 8. We find strong overlap between the genes found in the DE and DA analysis (roughly 78 genes), though scATAC-Seq is generally sparse with weaker signal than scRNA-Seq. Cumulatively these results support that clusters 13 and 16 are biologically distinct in both modalities with coherent signal between the two modalities, demonstrating that we are able to transfer finer resolution biological signal from the mRNA-Seq modality to the chromatin accessibility modality.*

Critique: "3. Cobolt aims to align cells from very distinct technologies and one concern for doing this is the batch effect across different platforms and datasets. Cobolt adjusts for batch effect by introducing a dataset specific B(i), however I feel that whether the batch correction works or not is not well evaluated in the case studies. The paper shows that the multi-modal data is helpful to better align the single-modal data by random splitting of the SNARE-seq cells. However, there is no batch differences here, so it would be nice if the authors can evaluate how robust Cobolt is when we anticipate batch effects across platforms."

*Response: To clarify, there are different terms in our model to deal with modality differences and batch effects. Specifically B(i) is the term that goes between the K dimensional space of our latent variable space to the di-dimensional space of modality i (e.g. from the latent space into into the gene expression space). This term in is the same for all datasets. We consider batch effects being due to being in different batches/platforms/datasets in the same modality i, such as mRNA expression from scRNA-Seq versus SNARE-Seq. The corrections for batch effect are actually in the expression of the sampling probabilities, π_c =σ(diag(α1(i))B(i)θ_c +α0(i)). The α0,α1 terms (per modality i and batch l combination) are the batch effect terms and provide scaling/centering before the soft-max transformation into probabilities.*

*Ultimately batch issues are going to be a serious concern for any kind of integrative analysis. We provide the ability to estimate batch effect terms inside the model, but we also envision that users may choose to use dedicated batch correction tools that have been developed specifically for the details of a particular modality. This is especially true for mRNA expression data, where the literature is quite mature. So long as the resulting output are batch-corrected counts, there is no problem using such data as input to our method. However, we also recognize that one of the interests is certainly for technologies with less developed batch-correction tools, and therefore we provide a mechanism for correcting for batch effects in our model.*

*We have added some additional analyses comparing the effectiveness of our batch correction terms, specifically how the analysis changes when we leave the α terms out and have just πc = σ(B(i)θc). As described above, with our 10X multiome data, we are able to have an earlier run of the 10X multiome data, which we used to evaluate the methods using our nearest neighbor approach (Fig 5) by treating this additional dataset as unpaired. Since these are run on different samples and at different times this also is a way of testing the performance of our batch correction terms. In Supplementary Figure 17 we show the improvement due to adding our batch correction. We also show in Supplmentary Figure 18 a qualitative UMAP visualization of the effect of our batch correction in the SNARE-Seq data, with and without the batch correction terms, which also show the correction term is improving the analysis. It is always difficult to have ground truth in evaluating batch effects, especially with emerging technologies, but we feel that this shows that our methods are effective at removing batch effects. As mentioned above, we would not be surprise to find that dedicated batch correction tools might offer some additional improvement, but our built-in technique seems to perform reasonably well.*

*We have included additional discussion and references to these additional supplementary figures in the Methods section when we discuss the batch correction terms.*

Critique: "4. For the variational autoencoder, based on my understanding, the authors assume that the posterior distribution of Z on cells with multimodal sequencing data (say (X(1),X(2))) is a product of the posterior distribution of Z on each of the observed modality (X(i)) (page 17). Will this hold only when marginally X(1) and X(2) are independent? So strictly speaking, is it true that the assumption on p(z|XA) (page 17 lines 21-23) is actually never satisfied? Anyway, I think the autoencoder structure of Coboit is a nice design."

*Response: This assumption requires the X(1) and X(2) to be independent conditional on the latent variable Z, but does not imply marginal independence of X(1) and X(2). (You can also see our response to Reviewer 2, question 1, regarding the related question of the assumption of conditional independence of X(1) and X(2) below.)*

Critique: "5. I’m still a bit confused about the assumption on p(X|Z). Seems that X are counts and authors assume that each entry of X follow a multinomial distribution. I do not quite understand why we can assume a multinomial distribution of X."

*Response: We assume that the vector of counts across all of the features follows a multinomial. In other words, of the N reads sequenced, the probability that a read came from feature of the platform (genes for mRNA or peak regions for ATAC-Seq) follows a multinomial. This is one of several standard models for modelling the entire vector of counts [Townes et al., 2019, Nowicka and Robinson, 2016].*

Critique: "6. What are the chosen values for the hyperparameters in equations S1 and S2?"

*Response: For Equation S1, we set the hyperparameter weights η for conditional likelihood terms to 1. We adopt a KL cost annealing schedule that linearly increases the weight of the KL term γ from 0 to 1 in the first 30 epochs. For Equation S2, we set the weighting terms λA reciprocal to the number of samples available for modality combination A. The above information can now be found in “Cobolt Network Architecture and Training” in Section 5.3.*

Critique: "7. There seems to be a typo on page 10 line 25, the last word should be “encoders” instead of “decoders”?"

*Response: Thank you for catching it. This typo has been fixed.*

Reviewer 2

Critique: "1) The author did not develop the original MVAE model, instead, they directly applied it on the task of integrate multiple single-cell multi-modal data without examining the property of MVAE and other VAE models designed for data of two or multiple modalities. This much lower the computational originality of this work. The advantage of MVAE is to handle datasets of a high number of modality, which have different level of missing measures on certain modalities. However, in single cell multi-omics data, normally joint analysis are only needed for two modalities (transcriptome and chromatin accessibility). There are several VAE models enabled the bidirectional generative models other than MVAE, such as JMVAE. The authors are expected to evaluate the most property model for single cell multi-omics data. Specifically, the MVAE assumes conditional in dependency of the modalities. However, this seems contradict to the underlying systems biology of transcriptional regulation. Gene expression and chromatin accessibility cannot be independent conditional to the low dimension latent variable z, which characterize the phenotypic characteristics of the cell, because chromatin accessibility is a very strong transcriptional regulatory signal that can directly affect the gene expression. Hence the conditional independence assumption of MVAE model could be fundamentally problematic for integrating scRNA-seq and scATAC-seq data."

*Response: We certainly did not develop the MVAE modeling framework, though we do adapt it for this setting in important ways (we have found that a direct application of the standard MVAE model performs quite poorly here because of various characteristics of the sequencing data). This is generally true of many computational biology methods, which frequently involve applying and adapting known algorithms in innovative ways so that they will perform well for the biological problem; this doesn’t invalidate their relevance.*

*We agree that there are many different VAE approaches that could have also been considered in this setting. Indeed the Babel algorithm, against which we compare, uses one of the bidirectional generative models that the reviewer suggests. Babel itself does not perform well on our data, though we have no reason to conclude that is because of the use of this model; but it does point to the difference between an intellectually appropriate underlying model and its actual performance in practice. The MVAE approach we use is also a reasonable choice in this setting, and we have shown that it is effective in modeling true biological signal.*

*In addition, we find the flexibility of the MVAE to expand beyond the case of just two modalities a strength. There are technologies that have more than two modalities (e.g. scNMT-seq [Clark et al., 2018] and scTrio-seq [Hou et al., 2016] ), and even more interesting is that there are many different technologies that do two modalities, with one of the modalities being mRNA expression, such as scMT-seq (gene expression and methylation) [Hu et al., 2016] and CITE-seq (transcriptome and epitope) [Stoeckius et al., 2017], that could be integrated together through our approach as well. We only mention this potential briefly in the manuscript as a discussion point, since we have not actually demonstrated its effectiveness in such a setting; indeed because these are new technologies, there are not as of yet many examples of these datasets on the same biological system appropriate for integration, though they are becoming more frequent all of the time. But it was important to us that Cobolt be well-positioned to expand to meet these new challenges. Most importantly, we have demonstrated that it performs well for the problem even when only considering the setting of two modalities.*

*With respect to the concern regarding conditional independence assumption of the MVAE, a variable X can be a strong predictor (including casually) of variable Y and there still exist a latent variable Z such that X and Y are conditionally independent of each other given Z. These are not contrary statements. In other words, chromatin accessibility can be a strong regulatory signal of gene expression, and there still exist a latent variable Z that sufficiently accurately predicts both of gene expression and chromatin status so that these variables are conditionally independent of each other when Z is known. For example, if chromatin status was the sole predictor of mRNA expression, then clearly a latent variable that recapitulated the chromatin status would be such a latent variable. The larger concern is whether our model is sufficiently rich enough to identify such a variable Z (in K dimensions) that has this property. We intentionally pick an extremely flexible model (MVAE), and we investigated different choices of K, but this is of course always a question in latent variable modeling. This is also a fundamental problem in all low-dimensional representations, generically, including standard tools such as PCA or UMAP, regardless of the context.*

Critique: "2) A few similar works were published recently:

Chunman Zuo, Luonan Chen, Deep-joint-learning analysis model of single cell transcriptome and open chromatin accessibility data, Briefings in Bioinformatics, Volume 22, Issue 4, July 2021, bbaa287 Wang, T., Shao, W., Huang, Z. et al. MOGONET integrates multi-omics data using graph convolutional networks allowing patient classification and biomarker identification. Nat Commun 12, 3445 (2021)."

*Response: Thank you for these references. Wang et al developed a supervised multi-omics integration method for biomedical classification, such as disease patient classification and cancer type classification. Therefore, it aims to solve different questions compared to \textit{Cobolt}, which is an unsupervised method.*

*The scMVAE algorithm from the work of Chunman et al is related to our work. However, like the MOFA2 algorithm, it does not incorporate single modality data in its analysis, but is a method to only analyze the multi-modality data. We have tried to run scMVAE through our comparisons on the SNARE-Seq data in Section 2.2. However, the code for scMVAE appears to have a bug in the code producing errors such that it will not run on our datasets. In looking at their source code, it appears to us that they have forgotten to convert one of their parameters to the log-scale, and after correcting this the method appears to run. We attached to this response a revised Figure 2 based on this corrected scMVAE code, showing that it doesn't appear to perform better either (see Figure 1C in figure provided in pdf verions). However, this is our diagnosis, and the authors of the package do not at this time think that this is the source of the bug. Thus we could not publish these results and portray them as coming from the scMVAE algorithm. Furthermore, we find the strength of Cobolt to be particularly in the integration of single-modality and multiple modality datasets, which scMVAE does not do. We have, however, included a citation to their work in the introduction.*

Critique: "3) VAE based models can hardly provide a deeper mining of biological functions. As presented in the manuscript, the current utilization of this method is only to improve cell clustering analysis. Cell clustering is a relatively well solved task in single cell data analysis. Mining of more biological insights are expected."

*Response: We would respectfully disagree that cell clustering is a solved task in this setting. The difficulty here is not the clustering, per se, but the representation and integration of all of these datasets into a single space such that clustering can take account of all of the cells irrespective of modality and platform. This is a non-trivial task that our algorithm solves. The joint representation can also be used in other downstream tasks that are typically usually done on estimated latent spaces, such as frequently provided by PCA on the analysis of a single-modality datasets. An important example is that of pseudo-time estimation; the integrated analysis of Cobolt allows for pseudo-time estimation to be constructed across cells from multiple modalities. We have mentioned this example in our discussion in the main text. However, it is even harder to have ground truth for pseudotime estimates than for clusters, so we have focused on the example of subtype identification for validating and comparing our methodology.*

*We have included a number of additional biological evaluations of the clusters we find with Cobolt in Section 2.2, including differential expression/accessibility analysis to find gene markers that validate their biological meaning. For example, we show that the small purple cluster in Figure 1A display strong markers of a layer 6 IT Car3 subset of cells, unlike the Layer 5/6 and Layer 2/3 cells surrounding it (Supplementary Figure 4). Similarly, the clusters found only in the mRNA in Supplementary figure S2A (now Supplementary Figure 1) show biomarkers for well-known non-neuronal cell types (astrocytes, microglial cells, oligodendrocyte cells, and oligodendrocytes precursors) as shown in the new Supplementary Figure S2. The green cluster found in chromatin in the Supplementary Figure S2B (now Supplementary Figure S1B) we show to have distinct chromatin accessibility peaks, including near the gene Car12 which is a marker gene of previously annotated subtype of Layer 6 (Supplementary Figure 2BC). We’ve similarly expanded our justification of the identifications of our clusters to Lamp5/Vip celltypes (Figure 4B and Supplementary Figure 8).*

**Second round of review**

**Reviewer 1**

The paper has been much improved after revision and the authors have addressed most of my comments. I only have two minor comments:

1. For my previous comment #4, I think I was confused about the form of the posterior p(z|X^A). In the supplement, the authors claim that the true posterior is proportional to \prod_i p(z|X^{i}), but I think we are unable to get this by only assuming that p(X^A|z) = \prod_i p(X^{i}|z). I feel that separation of the posterior of z would also need marginal independence across X^{i}

2. I'm not aware of methods that output batch-corrected counts. Seems to me that it's might be possible for a method to provide batch-corrected relative expression levels, but I can not think of a good way to return counts after batch correction. The authors may at least need a citation there. Also, if the input data are not counts after normalization, will Cobolt still be able to deal with that and provide good performance?

**Reviewer 2**

The authors have solved all my technical concerns.

**Authors Response**

**Point-by-point responses to the reviewers’ comments:**

*With respect to the comments of Review 1:*

*1. We have added in the Additional File 2: Supplementary Text an quick derivation of this result. In short, we use Bayes rule to rewrite p(z|X^A) in terms of p(X^A|z) which allows us to write the distribution in terms of \prod_i p(X^{i}|z) due to the conditional independence of the X^{i} given z. Another application of Bayes rule then converts this to be in terms of \prod_i p(z|X^{i}).*

*2. To be clearer, we were considering methods that return batch-corrected values that are either counts or on the *scale* of the original counts but could take decimal values, sometimes called pseudo-counts (as opposed to methods that provide basically residuals from a model after subtracting out effects due to batch). The output of some of these methods would have to be rounded to the nearest integer value (similar to transcript expression estimates from packages like Cufflinks). We have included references to some packages that provide such values.*
